# Immuno- and Constitutive Proteasomes Do Not Differ in Their Abilities to Degrade Ubiquitinated Proteins

**DOI:** 10.1016/j.cell.2013.01.037

**Published:** 2013-02-28

**Authors:** James A. Nathan, Valentina Spinnenhirn, Gunter Schmidtke, Michael Basler, Marcus Groettrup, Alfred L. Goldberg

**Affiliations:** 1 Department of Cell Biology, Harvard Medical School, Boston, MA 02115, USA; 2 Cambridge Institute for Medical Research, NIHR Cambridge Biomedical Research Centre, Addenbrooke’s Hospital Site, Cambridge CB2 0XY, UK; 3 Division of Immunology, Department of Biology, University of Konstanz, 78457 Konstanz, Germany; 4 Biotechnology Institute Thurgau at the University of Konstanz, 8280 Kreuzlingen, Switzerland

## Abstract

Immunoproteasomes are alternative forms of proteasomes that have an enhanced ability to generate antigenic peptides. Recently, Seifert and colleagues reported surprising observations concerning the functions of immunoproteasomes and cellular responses to interferon-γ: (1) that immunoproteasomes degrade ubiquitinated proteins faster than the constitutive proteasomes, (2) that polyubiquitin conjugates accumulate after interferon-γ treatment but then are preferentially degraded by immunoproteasomes, and (3) that immunoproteasome deficiency causes the formation of inclusions and more severe experimental autoimmune encephalomyelitis (EAE). In contrast, we find that polyubiquitin conjugates do not transiently accumulate following IFNγ-treatment and that immunoproteasomes do not prevent the formation of intracellular inclusions or protect against EAE. Furthermore, purified 26S constitutive and immunoproteasomes bind ubiquitin conjugates similarly and degrade them at similar rates. We conclude that, although immunoproteasomes can increase the generation of peptides appropriate for MHC class I presentation, they do not degrade ubiquitinated proteins more efficiently than constitutive particles.

## Highlights

► IFNγ treatment does not cause an accumulation of ubiquitinated proteins in cells ► Immuno- and constitutive proteasomes degrade ubiquitin conjugates at similar rates ► Immunoproteasomes do not prevent the formation of intracellular inclusions ► LMP7 null mice do not develop more severe experimental autoimmune encephalomyelitis

## Teaser

New studies challenge a previously published conclusion that immunoproteasomes degrade ubiquitinated proteins more rapidly than conventional proteasomes.

## Introduction

The presentation of intracellular peptides by MHC class I molecules at the cell surface is essential for immune surveillance, as well as for the detection of intracellular pathogens by marauding cytotoxic T cells (Groettrup et al., 2010). Protein breakdown by proteasomes generates the great majority of these peptides that are loaded onto the MHC class I complex (Rock and Goldberg, 1999; Rock et al., 1994). The constitutive 26S proteasome is a 2.5 megadalton complex composed of the 20S catalytic chamber, which contains the β subunits that catalyze peptide hydrolysis (β1, β2, and β5), and the 19S regulatory particle, which binds the polyubiquitinated substrates. The 19S base contains six ATPase subunits that catalyze protein unfolding, gate opening into the 20S core, and translocation into the central chamber of the 20S particle (Finley, 2009; Peth et al., 2010; Smith et al., 2011). After exposure of most cells to interferon-γ (IFNγ) or tumor necrosis factor (TNF)-α, special forms of the proteasome termed immunoproteasomes are expressed, in which the three catalytic subunits of the 20S are replaced by homologous subunits, β1i /LMP2, β2i/LMP10 (MECL1), and β5i/LMP7. In addition to this induction in inflammatory states, these alternative forms are actually found normally in immune tissues. Due to their distinct peptidase sites, they cleave proteins in a distinct manner from constitutive particles and generate more peptides capable of binding to MHC class I molecules, thereby serving an important role in antigen presentation (Gaczynska et al., 1993; Kincaid et al., 2012; Rock and Goldberg, 1999).

The immunoproteasome preferentially cleaves proteins after hydrophobic residues and less after acidic residues (Aki et al., 1994; Driscoll et al., 1993; Gaczynska et al., 1993, 1994), and these peptides with hydrophobic C termini are preferentially transported by TAP into the endoplasmic reticulum and loaded onto class I molecules. In vivo studies confirm that this change in the peptide repertoire presented at the cell surface is important for enhancing cytotoxic T cell responses (Basler et al., 2006; Fehling et al., 1994; Kincaid et al., 2012; Van Kaer et al., 1994).

20S immunoproteasomes exist in cells either as free particles, in association with the 19S regulator, (forming 26S immunoproteasomes), as well as in “hybrid 26S” with PA28αβ and the 19S regulator. Although the 20S immunoproteasome and the 20S constitutive proteasome have distinct cleavage site preferences, they degrade nonubiquitinated proteins at similar rates (Cascio et al., 2001), as do the two forms of the 26S proteasome. However, Seifert et al. (2010) recently reported evidence for additional intriguing roles of immunoproteasomes in the rapid breakdown of ubiquitinated proteins following cytokine exposure. These observations, if validated, would have important implications for understanding proteasomal mechanisms generally and the process of antigen presentation. They reported that IFNγ treatment caused a transient accumulation in cells of polyubiquitin conjugates, which they suggested was due to ubiquitination of newly synthesized proteins. Seifert et al. (2010) concluded that the accumulation of polyubiquitin conjugates results from a transient decrease in proteasome activity, while the cells are forming mature immunoproteasomes. In addition, they reported that immunoproteasomes are more efficient than constitutive particles in degrading polyubiquitinated proteins and are essential to remove damaged proteins in inflammatory states in mice, because they could efficiently digest misfolded proteins that form aggresome-like inclusions.

Such properties of immunoproteasomes would imply that this species of proteasome has a special capacity for eliminating ubiquitinated proteins, especially damaged proteins, as accumulate in multiple disease states and are often not efficiently degraded by the constitutive 26S particles. However, these proposed special functions for immunoproteasomes, which were further elaborated in the accompanying editorial (van Deventer and Neefjes, 2010), raises a number of fundamental questions that are difficult to resolve with our present understanding of proteasome function. In particular, it is unclear how would changes in the active site specificities of the core 20S particle alter its capacity to bind and degrade ubiquitinated proteins, which are functions of the 19S regulatory particles.

Because of their major implications for understanding proteasome mechanisms and inflammatory responses, our two laboratories have independently re-examined several of the key observations reported by Seifert et al. (2010), using both similar and alternative rigorous approaches. Our findings seriously challenge several of their key observations and the resulting models, as we find (1) that 26S immunoproteasomes have a similar capacity to bind and degrade polyubiquitinated proteins, as do constitutive proteasomes. This result supports the widely accepted view that the 19S regulatory complex (not the 20S core particle) is critical in determining their rates of degradation and (2) that in models of murine inflammation immunoproteasome deficiency has no effect on the formation of intracellular ubiquitin-containing inclusions or the in vivo manifestations of experimental autoimmune encephalomyelitis.

## Results

### IFNγ Does Not Cause an Accumulation of Polyubiquitin Conjugates during Induction of Immunoproteasomes

**Figure 1:**
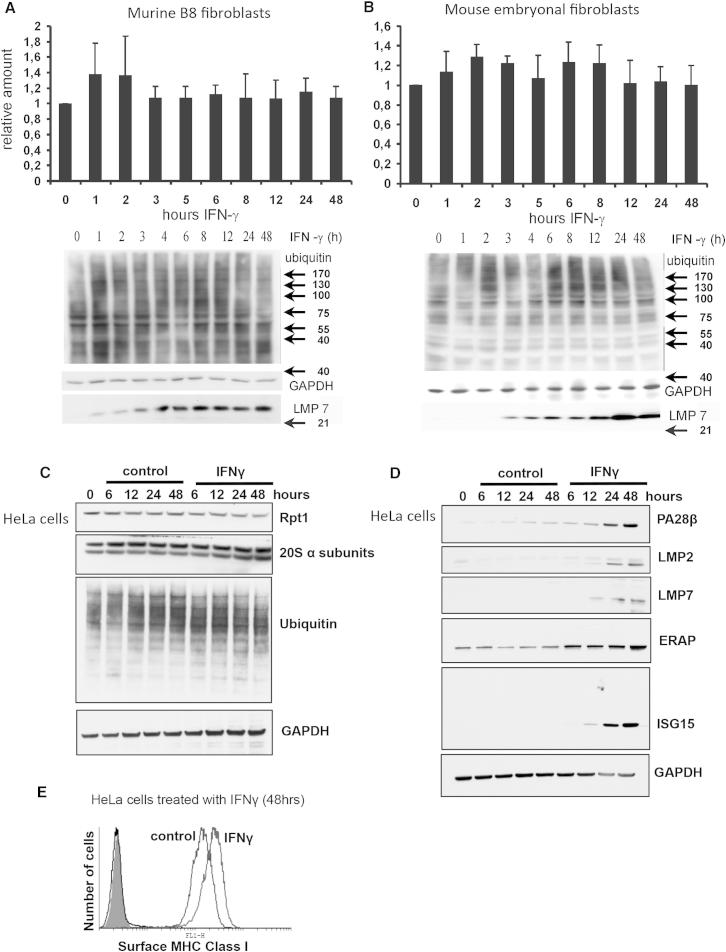
IFNγ Does Not Cause an Accumulation of Polyubiquitinated Proteins during the Induction of immunoproteasomes and Other Components of the MHC Class I Presentation Pathway (A and B) Western blot analysis and densitometric evaluation of polyubiquitin conjugates in mouse fibroblasts after IFNγ treatment. Lysates from the murine fibroblast cell line B8 (A) and embryonal fibroblasts of C57BL/6 mice (B) were analyzed for their content of ubiquitinated proteins by western blotting after exposure to IFNγ for the indicated times (A and B, bottom). GAPDH served as loading control. The amount of LMP7 increased, confirming the expected response to IFNγ stimulation. Graphical presentation of the means ± SEM of densitometric evaluation of the immunoblots (A and B, top) showed no significant accumulation of polyubiquitin conjugates after IFNγ stimulation (Student’s t test). (C–E) Treatment of HeLa cells with IFNγ does not lead to a transient accumulation of polyubiquitin conjugates. HeLa cells were treated with or without 100 U/ml IFNγ for the indicated times. The cells were lysed by sonication and centrifugated at 100,000 × *g* for 1 hr. The levels of the ubiquitin conjugates (C), proteasome subunits (C), and proteins known to be involved in the IFNγ response (D) were analyzed by western blotting. (E) IFNγ treatment increased surface MHC class I expression. A proportion of the HeLa cells (2 × 10^5^) that were treated with or without IFNγ for 48 hr (C) were incubated with a FITC conjugated conformational MHC class I antibody (W6/32), and the levels of cell surface class I molecules were measured by flow cytometry. See also Figure S1.

To determine the influence of IFNγ stimulation on the cellular content of polyubiquitin conjugates, we treated the BALB/c-derived fibroblast cell line, B8, and freshly prepared mouse embryonal fibroblasts (MEFs) of C57BL/6 mice with IFNγ (200 U/ml). At the indicated time points, aliquots were taken, lysed in the same manner as used by Seifert et al. (2010), and analyzed by western blotting with antibodies against polyubiquitin and GAPDH (as a loading control). Although one of us had previously observed an increase in polyubiquitin conjugates in IFNγ-treated HeLa cells, in these experiments with B8 cells, in contrast to the data in Seifert et al. (2010), no significant increase in high molecular weight ubiquitin conjugates was detected in either cell line during the 48 hr after IFNγ treatment (Figures 1A and 1B). In particular, we did not observe any transient increase in ubiquitin conjugates as was reported by Seifert et al. (2010) in cell line B8, 4–12 hr after IFNγ addition. To confirm that the cells were responding to IFNγ, we measured the induction of the immunoproteasome subunit LMP7 (Gaczynska et al., 1993; Rock and Goldberg, 1999), which typically increases following interferon exposure. By 6 hr exposure to IFNγ, there was an elevated level of the functional (fully processed) LMP7 (23 kDa), which results from proteolytic processing of the 30 kD LMP7 precursor during the assembly of immunoproteasomes. Because this induction of immunoproteasomes occurred without a reduction in the amounts of polyubiquitin conjugates, there was no experimental basis for the suggestion (Seifert et al., 2010) that these particles have a special capability to degrade ubiquitin conjugates.

**Figure S1:**
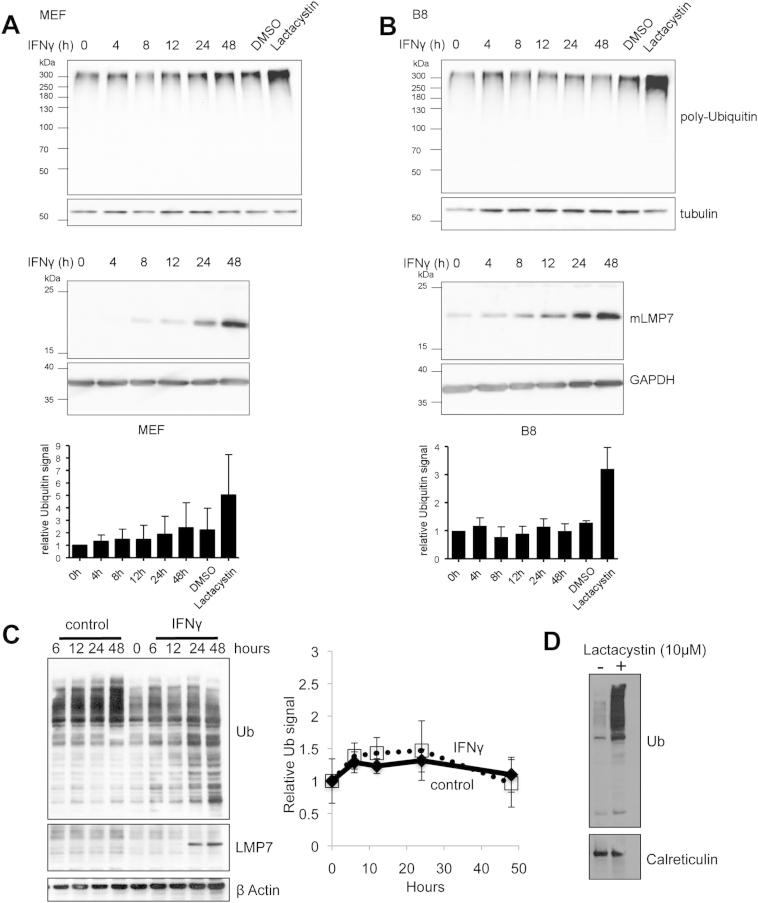
IFNγ Treatment Does Not Cause a Significant Accumulation of Polyubiquitin Conjugates during the Induction of Immunoproteasomes, Related to Figure 1 (A and B) Murine embryonic (A) and B8 (B) fibroblasts were stimulated with 100U/ml IFNγ for the indicated time periods. As a control for ubiquitin accumulation, cells were treated with the proteasome inhibitor lactacystin (10μM, 4hr) or vehicle (DMSO). The cells were then lysed by sonication in 25mM HEPES pH 7.4, 1mM ATP, 1mM DTT, 5mM MgCl_2_ and 10% glycerol. Lysates were subsequently centrifuged for one hour at 100,000 x g. Protein concentrations of the lysates were determined using the DC Protein Assay (Biorad) and equal amounts of protein were separated by SDS-PAGE and analyzed by western blotting. α-tubulin served as a loading control. One representative experiment out of three independent experiments with similar outcomes is shown. The induction of LMP7 by IFNγ stimulation was confirmed by immunoblot analysis; GAPDH served as a loading control. The ubiquitin levels were determined by densitometric analyses (ImageJ) of five different western blots; shown are the mean values ± SD obtained after normalization to the loading control and relative to the value for unstimulated cells which was set to unity. (C and D) HeLa cells were treated with 100U/ml IFNγ as described (C) or with 10 μM lactacystin for 4 hr (D). The cells were lysed with nonionic detergent according to the methods described by Seifert et al. (20 mM TRIS-HCl, pH 7.5, 10 mM EDTA, 100 mM NaCl, 1% NP40, 10 μM MG-132, 5 mM NEM and Complete Protease Inhibitor Cocktail (Roche)) and immunblotted for ubiquitin (C, left). The ubiquitin levels, (relative to the β-actin control) were determined by densitometric analyses (ImageJ) as described (C, right). The mean values ± SEM for three replicates are shown (C).

In these experiments, cells were disrupted by use of a nonionic detergent, which may extract both soluble polyubiquitin conjugates and ubiquitin containing protein aggregates. We therefore used a more stringent method to isolate only these soluble polyubiquitinated proteins that might be degraded by the 26S. Following the IFNγ treatment, the MEFs were lysed by sonication, and the soluble fraction isolated by 100,000 × *g* centrifugation (Figures S1A and S1B available online). Again, no significant change in the levels of polyubiquitin conjugates was detected after IFNγ stimulation (Figures S1A and S1B). Thus, we could find no basis for the conclusion that polyubiquitin conjugates transiently increase after IFNγ treatment.

To further explore if IFNγ stimulation could be seen to cause an accumulation of polyubiquitinated proteins, these experiments were repeated using a different cell line and in another laboratory. HeLa cells were treated with IFNγ (100 U/ml), and the levels of high molecular weight polyubiquitin conjugates measured following cell disruption by sonication and extraction of the soluble fraction (Figure 1C) or after lysis with nonionic detergent (Figures S1C and S1D). These HeLa cells had responded to the IFNγ because immunoproteasome subunits and other components of the MHC class I presentation pathway, which typically increase following IFNγ exposure, were all clearly induced. Twelve hours after the IFNγ addition, LMP2 and LMP7 subunits of the immunoproteasome were induced, as well as PA28αβ and ER-associated peptidase 1 (ERAP1), which trims longer proteasome products to eight to ten residue-peptides for efficient loading onto the MHC class I complex (Chang et al., 2005; Saric et al., 2002) (Figure 1D). Furthermore, flow cytometry analysis of surface MHC class I expression showed that there was a marked increase of class I molecules at the plasma membrane at 48 hr, indicating a normal IFNγ response in these cells (Figure 1E). Interestingly, in these cells, the total content of 26S and 20S particles, as measured by the 19S component, Rpn1, and the 20S α-subunit levels, were not altered (Figure 1C). Despite this clear induction of the IFNγ-responsive genes, there was, no reproducible change in levels of polyubiquitin conjugates (Figures 1C and S1C). Thus despite trying different lysis protocols and different cell lines, in these experiments we were not able to reproduce the previously reported accumulation of polyubiquitin conjugates upon immunoproteasome induction.

Seifert et al. (2010) also reported that the ubiquitin conjugating enzyme, Ube2L6 (also known as UbcH8), is induced following IFNγ treatment and suggested that this E2 is required for the accumulation of polyubiquitin conjugates. One concern regarding this conclusion is that it is now well established that Ube2L6, although able to facilitate protein ubiquitination, primarily functions in protein modification by ligation of the ubiquitin-like protein, ISG15 (Zhao et al., 2004). ISG15 is known to be induced by type I interferons IFNα and β rather than type II interferons (IFNγ) (Zhao et al., 2005). Therefore, we tested whether ISG15 levels and protein conjugates are also increased in the HeLa cells following IFNγ exposure. Surprisingly, we found that monomeric ISG15 was induced with IFNγ, although we could not detect ISG15 conjugates (Figure 1D). It is currently unclear why ISG15 increases in response to IFNγ, but the monomeric form may have an antiviral function (Skaug and Chen, 2010). However, it is noteworthy that Seifert et al. (2010) found that the E3 for ISG15, Herc5, was also induced, together with Ube2L6, strongly suggesting an increased capacity for ISG15 ligation to proteins.

### Proteasome Activity Does Not Transiently Decrease in Response to IFNγ

**Figure 2:**
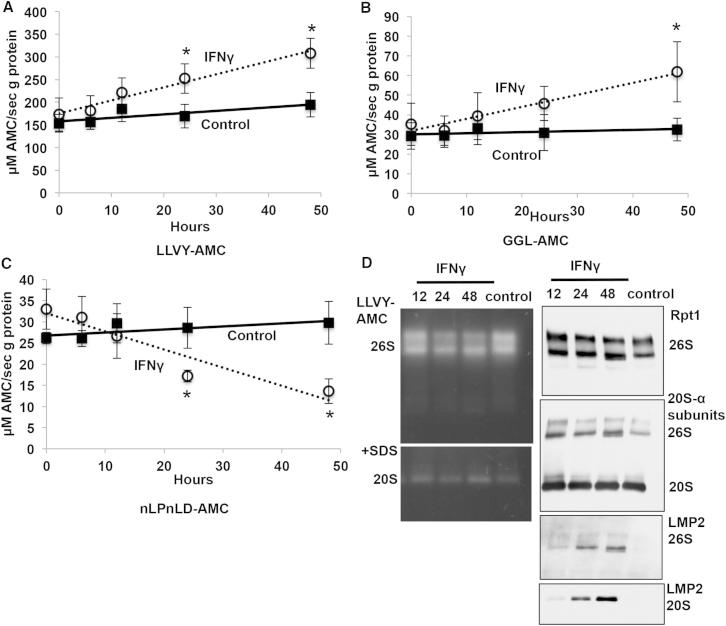
Proteasome Activity Does Not Transiently Decrease in Response to IFNγ (A–C) Following treatment with IFNγ, the chymotrypsin-like activity of the 26S proteasome increased progressively, whereas the caspase-like activity decreased. HeLa cells were treated with or without 100 U/ml IFNγ for the indicated times. The proteasomal peptidase activity was then measured in the lysates using Suc-LLVY-AMC and Suc-GGL-AMC for the chymotrypsin-like activity, and Ac-nLPnLD-AMC for the caspase-like activity. The lysates were also treated with 1 μM Bortezomib/Velcade to determine the Bortezomib-resistant (nonproteasomal) activity, which was subtracted from the total activity at each time point to measure only the proteasomal activity. All values are means of three separate experiments ± SEM. ^∗^p < 0.05. (D) Native PAGE analysis of the lysates from cells treated with IFNγ in (A)–(C). Lysate protein (25 μg) was separated using 3%–8% Tris-acetate gels containing 1 mM ATP and 5 mM MgCl_2_. In-gel proteasome activity was measured by incubating the gels in a Tris buffer containing 1 mM ATP, 5 mM MgCl_2_, and 50 μM Suc-LLVY-AMC at 37°C for 30 min (D, upper-left). The activity of the 20S proteasomes was visualized by adding 0.02% SDS for 45 min (D, lower-left). Proteasome complexes were visualized by western blotting for the 19S regulator (Rpt1), 20S proteasomes (α subunits), and immunoproteasome subunit LMP2 (D, right panels). The 26S preparations contain both doubly (19S-20S-19S) and singly (19S-20S) capped species and there was no significant change in the levels of these complexes following IFNγ treatment.

Although the total levels of ubiquitin conjugates did not change, it remained possible that proteasomal activity had increased in response to IFNγ. To examine this possibility, HeLa cells were treated with IFNγ for up to 48 hr, and lysates isolated in the usual manner. The total proteasomal peptidase activities in the HeLa extracts were measured by assaying cleavage of specific substrates of the chymotrypsin-like site, Suc-LLVY-AMC and Suc-GGL-AMC, and of the caspase-like site, Ac-nLPnLD-AMC. To ensure that their hydrolysis was only due to proteasomes, the lysates were treated with the specific proteasome inhibitor, Bortezomib (1 μM), and the Bortezomib-insensitive activity subtracted from the total peptidase activity. The proteasome’s trypsin-like activity cannot be assayed in crude extracts due to the high nonproteasomal activity against its standard substrates (Kisselev and Goldberg, 2005). By 12 hr after IFNγ addition, there was an increase in the proteasome’s chymotrypsin-like activity and a decrease in its caspase-like activity (Figures 2A–2C), which coincided with the appearance of the immunoproteasome subunits (Figure 1D). These studies thus confirm the changes in peptidase activity previously reported upon induction of immunoproteasomes (Aki et al., 1994; Driscoll et al., 1993; Gaczynska et al., 1993, 1994) (Figures S1E–S1G). Importantly, we did not observe any transient decrease in proteasomal peptide-hydrolysis, as reported (Seifert et al., 2010).

To further confirm that proteasome content did not decrease in response to IFNγ, we analyzed the proteasome composition in the total cell lysate. HeLa cells were treated with IFNγ and lysed in the usual manner. Native PAGE was used to fractionate the different proteasome complexes, and we measured by western blotting, the 19S regulator subunit Rpt1 and α-subunits of the constitutive 20S proteasome, as well as proteasome activity using in-gel Suc-LLVY-AMC hydrolysis. In accord with our previous findings (Figure 1), there was no significant decrease in proteasome content or activity (Figure 2D). Instead, the levels of doubly and singly capped 26S, as well as free 20S proteasomes remained constant following IFNγ treatment.

Western blot analysis with an anti-LMP2 antibody confirmed that the catalytic subunits of the immunoproteasomes were first incorporated into mature 26S proteasomes by 12 hr, which coincided exactly with the first observed change in proteasomal peptidase activity in the lysates (Figures 2A–2C). Moreover, by 48 hr, both the presence of LMP2 and the changes in peptidase activity were more pronounced. Therefore, although immunoproteasomes were clearly generated, there was no evident change in overall proteasome content and no decrease in proteasome capacity. These findings and the lack of changes in ubiquitin conjugate levels also do not support the conclusion that 26S immunoproteasomes are more efficient than constitutive particles in degrading polyubiquitinated proteins (see below).

### Purified Immuno- and Constitutive 26S Proteasomes Degrade Ubiquitin Conjugates Similarly

**Figure S2:**
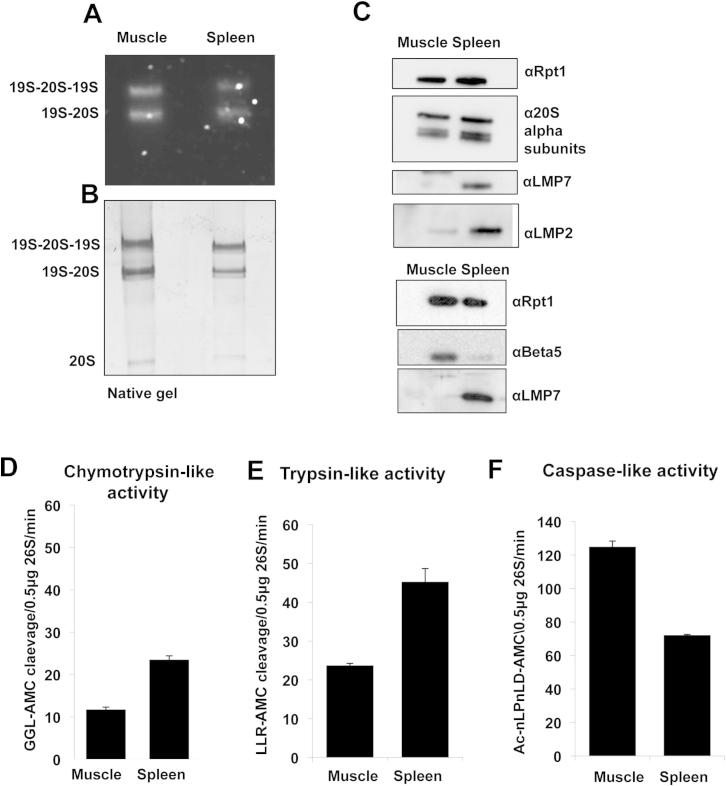
26S Immuno- and Constitutive Proteasome Preparations Show Similar Amounts of Doubly and Singly Capped Species but Differ in Their Peptidase Activities, Related to Figure 3 (A–C) 26S immunoproteasomes and 26S constitutive proteasomes from CD1 mice show comparable levels of doubly and singly capped proteasome particles. Constitutive 26S proteasomes were isolated using the Ubl-method from muscles and immunoproteasomes from spleens of CD1 mice. (A) In-native gel LLVY-AMC cleavage. (B) Silver-stained native PAGE analysis of the samples in (A). (C) Immunoblot of the 19S regulator subunit Rpt1 and 20S core subunits (beta5 for the constitutive 26S proteasome, and LMP2 and LMP7 for 26S immunoproteasomes). (D–F) Constitutive and immunoproteasomes differ in their peptidase activities. The pure proteasomes were incubated with the indicated peptides and the peptidolytic activity was measured by determining the fluorescence of the AMC leaving group. Shown are (D) chymotrypsin-like, (E) trypsin-like, and (F) caspase-like activities. All values represent means ± SEM.

To test this conclusion more directly, we purified 26S immuno- and constitutive proteasomes and compared their abilities to bind and degrade polyubiquitinated substrates. Prior studies showed that skeletal muscles contain only constitutive proteasomes, whereas spleens largely contain immunoproteasomes (Cascio et al., 2001; Van Kaer et al., 1994). Therefore, constitutive 26S particles were affinity-purified from muscles of CD1 mice and the immunoproteasomes from their spleens as described by Besche et al. (2009). To ensure that the total proteasome content was the same for each type, we analyzed the compositions of the purified proteasomes by SDS- and native PAGE. The ratios of the doubly to singly capped 26S species were comparable (Figures S2A–S2C), and as expected, the characteristic immunoproteasome subunits were present only in the particles from the spleens (Figure S2C). Also, the immunoproteasomes had approximately 2-fold greater chymotrypsin and trypsin-like activities, but a 2-fold lower caspase-like activity (Figures S2D–S2F).

**Figure 3:**
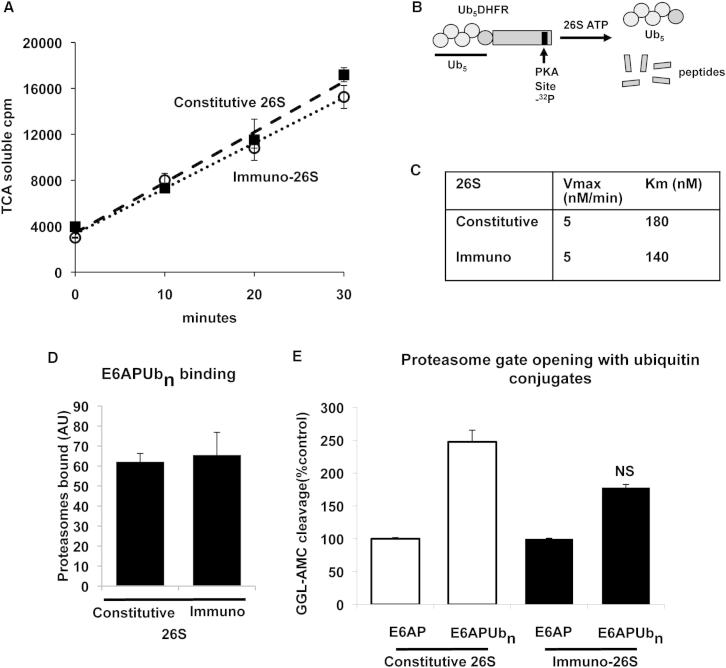
Immunoproteasomes and Constitutive 26S Proteasomes Degrade Polyubiquitinated Conjugates at Similar Rates Constitutive 26S proteasomes were isolated from muscles and immunoproteasomes from spleens of CD1 mice using the Ubl method (Besche et al., 2009). The subunit content of the different proteasome species, and their peptidase activity are shown in Figure S2. (A–C) The 26S constitutive proteasome and 26S immunoproteasome degrade Ub_5_DHFR at identical rates. The proteasome substrate, ^32^P-Ub_5_DHFR (B), was incubated at 37°C for 30 min with the pure proteasomes isolated from muscles and spleens of CD1 mice. Degradation of the ^32^P-Ub_5_DHFR to acid-soluble peptides was measured after precipitation of the samples with TCA (A). To calculate the K_m_ and V_max_ values, the ^32^P-Ub_5_DHFR was diluted with increasing concentrations of unlabeled Ub_5_DHFR (C). All values are means of three independent experiments ± SEM. NS, not significant. (D) 26S constitutive and immunoproteasomes bind ubiquitin conjugates similarly. The pure 26S proteasomes were incubated with resin-bound ubiquitinated E6AP (E6APUb_n_) at 4°C, washed, and the proteasomes bound to the conjugates were measured (Peth et al., 2010). (E) Ubiquitinated conjugates stimulate gate-opening similarly in the 26S constitutive and immunoproteasome. The 26S proteasomes were incubated with E6AP or E6APUb_n_, 1 mM ATP, and 5 mM MgCl_2_ at 37°C. Gate-opening was measured by the increase in peptide hydrolysis using Suc-GGL-AMC (Peth et al., 2009, 2010). See also Figure S2.

Because Seifert et al. (2010) had reported a greater capacity of the immunoproteasomes to degrade ubiquitinated substrates, we compared the rates of degradation by the constitutive and immunoproteasome species of a homogeneous polyubiquitinated substrate, ubiquitinated dihydrofolate reductase (Ub_5_DHFR). This substrate contains four lysines linked in a K48 chain to a ubiquitin on the N terminus of DHFR and encodes a protein kinase A (PKA) phosphorylation site near its C terminus. The ubiquitinated DHFR could therefore be radiolabeled using ^32^P-ATP, allowing the precise monitoring of its degradation by measuring the generation of TCA-soluble peptide fragments (Figures 3A–3C) (Lam et al., 2005). Degradation of this ubiquitinated DHFR was blocked by proteasome inhibitors or methotrexate, which prevent the degradation of the DHFR both by pure 26S (Peth et al., 2009) and in cells (Johnston et al., 1995). The purified 26S proteasomes from the mouse muscles and spleens were incubated with the radiolabeled Ub_5_DHFR and ATP at 37°C for up to 30 min. The ^32^P-Ub_5_DHFR was degraded by the isolated constitutive and immunoproteasomes at virtually the same rate (Figure 3A). To compare their degradative capacities more precisely, we incubated them with increasing concentrations of nonradiolabeled Ub_5_DHFR added and a constant amount of the ^32^P-Ub_5_DHFR, The Michaelis-Menten analysis showed no significant difference in their K_m_ (140 nM spleen, 180 nM muscle) or V_max_ (5 nM/min spleen, 5 nM/min muscle) for Ub_5_DHFR degradation by the constitutive or immunoproteasomes (Figure 3C).

To examine whether these proteasome species might bind ubiquitin conjugates more efficiently, we used a resin-bound polyubiquitinated protein as an affinity ligand (Peth et al., 2010). Lysine-48 polyubiquitin conjugates were formed by incubating the ubiquitin ligase, E6AP, (bound to a GST resin), with E1, E2, ubiquitin, and ATP (Kim et al., 2007; Peth et al., 2009). These conjugates were then incubated with the pure muscle or spleen 26S proteasomes at 4°C, and the amounts bound were measured (Peth et al., 2010). As shown in Figure 3D, there was no difference in the abilities of the constitutive and the immuno-26S-proteasomes to bind to polyubiquitin chains.

Once a polyubiquitin chain binds to the 19S component of the proteasome, they stimulate peptide hydrolysis by binding to the deubiquitinating enzyme, USP14, and by enhancing opening of the gated channel into the 20S particle (Peth et al., 2009). We therefore measured the ability of ubiquitin conjugates to activate peptide hydrolysis (Peth et al., 2009) in both forms of the 26S proteasomes. When pure 26S proteasomes and immunoproteasomes were incubated with E6AP or polyubiquitinated E6AP, the ubiquitin conjugates increased peptide hydrolysis about 2-fold in both the constitutive and immuno- 26S particles (Figure 3E). Thus, no evidence could be found by any of these approaches for the claim by Seifert et al. (2010) that immunoproteasomes are more efficient in degrading ubiquitinated proteins, and thus, no evidence that immunoproteasomes alter substrate processing by the 19S complex.

### LMP7 Deficiency Does Not Affect Formation of Inclusions or Polyubiquitin Conjugates in Mouse Embryonic Fibroblasts

**Figure 4:**
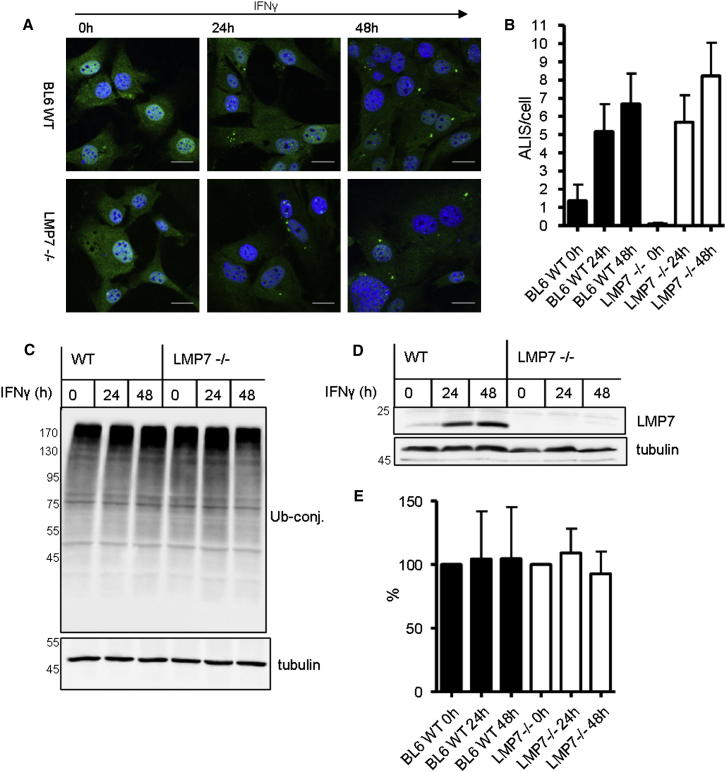
LMP7 Deficiency Does Not Affect Formation of Ubiquitin-Containing Inclusions and Total Soluble Ubiquitin Conjugates in MEF Cells (A) Formation of ubiquitin containing inclusions (ALISs) was visualized with a LSM510 confocal laser-scanning microscope (Carl Zeiss) after staining MEF cells with the ubiquitin-specific mAb FK2 (green). In MEFs from *LMP7*^*−/−*^ and C57BL/6 wild-type mice (BL6WT), ubiquitin was detectable in ALIS after IFNγ exposure for the indicated times. MEFs from LMP7 deficient and wild-type mice showed similar kinetics of ALIS formation. Scale bar, 20 μm. (B) Statistical evaluation of the number of ALIS per cell was performed for three independent experiments ± SEM (p value = 0.54 for 48 hr data; N > 210 cells). The images were analyzed with ImageJ software. (C) Western blot analysis of ubiquitin conjugates in MEFs from *LMP7*^*−/−*^ and C57BL/6 wild-type mice at different times after exposure to IFNγ; α-tubulin served as loading control. One representative experiment out of three independent experiments with similar outcomes is shown. (D) The induction of LMP7 by 200U/ml IFNγ stimulation was confirmed by immunoblot analysis; α-tubulin served as loading control. (E) Graph showing the ubiquitin levels determined by densitometric analyses of four different western blots of the kind shown in (C); shown are the mean values ± SD (p value = 0.61 for 48 hr data points) obtained after normalization to α-tubulin and relative to the value for C57BL/6 wild-type mice before IFN-γ stimulation (BL6 WT 0 hr) that was set to 100%. No statistically significant enhancement of ubiquitin conjugates after IFNγ treatment and also no significant difference between MEFs from WT and *LMP7*^*−/−*^ mice was found.

Under stressful conditions where misfolded proteins accumulate in cells, such as upon treatment with proteasome inhibitors, all cells form inclusion-like structures (termed aggresomes or aggresome-like-induced structures [ALIS]) that contain polyubiquitinated proteins (Szeto et al., 2006). Seifert et al. (2010) reported that the formation of such inclusions are induced by IFNγ due to a transient decrease in proteasome activity during the shift from 26S constitutive to immunoproteasomes. Because we found no evidence of such a decrease in proteasome activity following IFNγ exposure, we tested whether, in fact, inclusions are formed differently in mouse embryonal fibroblast (MEF) cells generated from wild-type and *LMP7*^*−/−*^ mice. MEF cells from C57BL/6 wild-type and *LMP7*^*−/−*^ mice were grown on coverslips and treated with IFNγ (200 U/ml). Formation of ubiquitin-containing inclusions was measured by confocal microscopy of large numbers of images. As reported by Seifert et al. (2010), we could also detect inclusion formation after IFNγ treatment in both wild-type and *LMP7*^*−/−*^ MEFs (Figure 4A). However, in contrast to their data, we found that the number of aggregates per cell increased to the same extent in LMP7-deficient and wild-type MEFs (Figure 4B). This increase in inclusions is presumably due to accumulation of aberrant proteins through de novo production or damage. In any case, because the loss of LMP7 did not enhance inclusion formation, the constitutive and immunoproteasomes seem to have similar capacities in vivo to eliminate such ubiquitinated proteins, in accord with the findings on pure proteasomes.

Furthermore, when we analyzed the levels of polyubiquitin conjugates in MEFs from these wild-type and *LMP7*^*−/−*^ mice by western blotting, in contrast to the findings by Seifert et al. (2010), we again observed no transient accumulation of ubiquitinated proteins in the wild-type cells after addition of IFNγ, which did induce LMP7 (Figure 4D). At first sight, it may seem contradictory that the number of inclusions that contain ubiquitin conjugates increases in MEFs after IFNγ treatment, even though the amount of polyubiquitinated proteins remains unchanged. However, as noted by Seifert et al. (2010), the nonionic detergents NP-40 or Triton X-100 were used for lysis and will not solubilize these aggregates. In addition, there was no difference in the total amount of polyubiquitin conjugates in MEFs from wild-type and *LMP7*^*−/−*^ mice 24 and 48 hr after IFNγ treatment (Figure 4C) as confirmed by quantitative densitometric analysis (Figure 4E).

### LMP7-Deficient Mice Are Not More Susceptible to Experimental Autoimmune Encephalomyelitis

**Figure 5:**
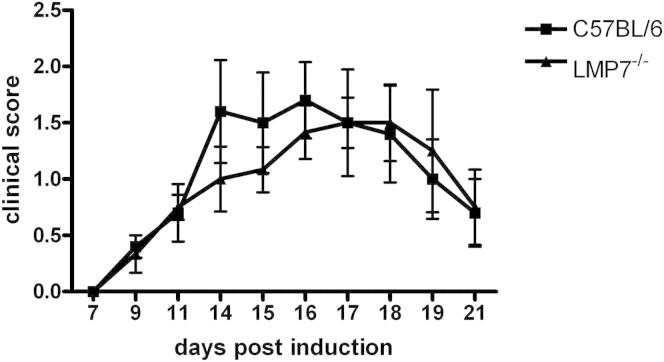
LMP7-Deficient Mice Are Not More Susceptible to Experimental Autoimmune Encephalomyelitis Than Wild-Type Mice C57BL/6 wild-type and *LMP7*^*−/−*^ mice were immunized with MOG_35-55_ peptide. Mice were daily monitored for clinical symptoms of experimental autoimmune encephalomyelitis (EAE). Symptoms were scored as: 0, no detectable signs of EAE; 0.5, distal limp tail; 1, complete limp tail; 1.5, limp tail and hind limb weakness; 2, unilateral partial hind limb paralysis; 2.5, bilateral partial hind limb paralysis; 3, complete bilateral hind limb paralysis; 3.5, complete hind limb paralysis and unilateral forelimb paralysis; 4, total paralysis of fore and hind limbs; 5, death. (y axis) is plotted versus time postimmunization (x axis). Data represent means ± SEM of six mice. The experiments were performed three times, yielding similar results.

Because our findings do not support an increased capacity of immunoproteasomes to degrade polyubiquitinated proteins, we also investigated the proposed special role of immunoproteasomes in enhancing the cell’s ability to combat systemic inflammatory conditions. We therefore examined the in vivo effects of LMP7 deficiency using the same model of multiple sclerosis used by Seifert et al. (2010), experimental autoimmune encephalomyelitis (EAE). *LMP7*^*−/−*^ and C57BL/6 control mice were immunized with MOG_35–55_ peptide to induce EAE. The disease scores of the mice were recorded in a blinded manner for 21 days. No significant difference in disease scores was observed between *LMP7*^*−/−*^ and wild-type mice (Figure 5), in clear contrast to the results reported by Seifert et al. (2010) but consistent with the study by Frausto et al. (2007), who found no effect of LMP2 deficiency on the course of EAE. Thus, the immunoproteasome does not have a protective effect against the development of EAE-induced inflammation.

## Discussion

It is now well established that the presence of immunoproteasomes is important for efficient MHC class I presentation and cytokine production (Groettrup et al., 2010; Kincaid et al., 2012), which accounts for their presence in white blood cells and induction during inflammation. Furthermore, the altered peptidase activity of the immuno-β-subunits can nicely account for their ability to generate an altered antigen repertoire. The findings of Seifert et al. (2010) indicated that in addition, immunoproteasomes have an enhanced capacity to degrade polyubiquitinated conjugates, which would mean that somehow the active sites in the central chamber of the 20S core particle alter the ability of the 19S to bind and process ubiquitinated proteins. However, by several distinct approaches, we failed to observe any such differences using both cellular and biochemical assays on extracts or pure 26S particles. Although IFNγ treatment did increase the amount of ubiquitin-containing inclusions in the cells, as Seifert et al. (2010) reported, there was no difference in their levels in immunoproteasome-deficient MEFs. Furthermore, using the same lysis conditions as Seifert et al. (2010), or our more stringent method of isolating the soluble fraction, the total levels of polyubiquitin conjugates in cell lines (HeLa) or primary cells (MEFs) remained unchanged after IFNγ exposure. How or why polyubiquitinated proteins accumulate in inclusions following IFNγ treatment is not clear and both are interesting questions for further study. These inclusions presumably contain damaged polyubiquitinated proteins, resembling aggresomes, perhaps due to free-radical damage to cell proteins as Seifert et al. (2010) suggest. However, their accumulation clearly is not due to changes in total cell proteasome content, which remains constant. Ubiquitin-containing inclusions are also observed following oxidative stress and can contain both short- and long-lived proteins (Kopito, 2000; Szeto et al., 2006). These inclusions can be cleared by both proteasomal and autophagy pathways (Szeto et al., 2006), but immunoproteasomes do not appear to have any distinctive role in this process.

Our experiments provide further evidence that the changes in proteasome peptidase activity following IFNγ exposure reflects the formation of the immunoproteasomes and their altered cleavage specificities, and is not due to any decrease or increase in total proteasome capacity. Measurements of a single peptidase activity to proteasome function can be misleading. As Seifert et al. (2010) measured only the chymotrypsin-like activity in the lysates (that increases selectively with incorporation of the immune-β-subunits), it is not possible to conclude that proteasomes are “more active” following IFNγ treatment. Any such analysis must also take into account the “caspase-like” site, which in immunoproteasomes is less active against acidic substrates and cleaves more after large hydrophobic residues (Rock and Goldberg, 1999). In fact, we found no decrease in proteasome content after IFNγ exposure, and instead, there is the steady maturation and increase in 26S immunoproteasomes.

The present studies used several newly developed quantitative assays for 26S function including the accurate kinetic measurement of breakdown of a ubiquitinated protein, Ub_5_DHFR, which until recently had not been possible, as well as ubiquitin conjugate binding to the 26S proteasome and the ability of ubiquitinated proteins to activate peptide hydrolysis by the 26S proteasome. In addition, we also measured the levels of 20S proteasomes, and the singly and doubly capped 26S particles, which is important for rigorous comparisons of the degradation of ubiquitinated proteins by the immuno- and constitutive 26S. Our demonstration that these particles bind and degrade ubiquitinated proteins at similar rates is consistent with prior findings that the 20S and 26S immunoproteasomes degrade denatured ovalbumin at the same rates as the corresponding constitutive particles (Cascio et al., 2001). Furthermore, immunoproteasome deficient mice, which lack the LMP2, 7, and 10 catalytic subunits, show clear defects in presenting MHC class I epitopes, but no difference in the total levels of polyubiquitin conjugates measured by immunoblot (Kincaid et al., 2012). As polyubiquitinated proteins bind initially to the 19S ubiquitin receptors Rpn10 and Rpn13 subunits, which do not differ between the immuno- and constitutive 26S, it is not surprising that they degrade ubiquitin conjugates similarly. In addition, the recent structural analysis of the binding surfaces for the 19S regulator both of the immuno- and constitutive 20S proteasome shows high similarity, such that the gating mechanisms for substrate entry are unlikely to differ (Huber et al., 2012). In summary, the complimentary efforts of our two labs yielded no evidence that ubiquitinated proteins are degraded faster by the immuno-26S proteasomes.

The roles of the other proteasome regulators following IFNγ exposure (e.g., PA28αβ) on protein degradation is less clear. Although PA28 is induced, its precise functions in antigen presentation and perhaps in other proteasomal processes (Pickering et al., 2010) are still unclear. Hybrid proteasomes (19S-20S-PA28) have been shown to generate a different peptide repertoire compared to the constitutive 26S proteasome, but show no increase in the rate of protein breakdown (Cascio et al., 2002). PA28αβ has also been suggested to increase the ubiquitin-independent degradation of oxidatively damaged proteins, although biochemical evidence on PA28’s specific role and mechanism are still lacking (Pickering et al., 2010). Further studies are required to clarify how PA28αβ promotes immune surveillance in response to IFNγ and degradation of specific types of damaged proteins, and whether as suggested (Seifert et al., 2010; van Deventer and Neefjes, 2010), these seemingly distinct roles are linked.

The results of Seifert et al. (2010) have been used to propose a model of immunoproteasome function in which newly transcribed proteins are damaged by IFNγ-induced free radicals, leading to increased proteasomal degradation and MHC class I presentation, as well as the formation of ubiquitin-containing inclusions (van Deventer and Neefjes, 2010). We have not found any evidence that immunoproteasomes have a greater capacity for degradation of ubiquitinated proteins, and we have also failed to confirm other aspects of this proposed model. There is presently no evidence that MHC class I peptides are derived from newly synthesized, oxidatively damaged proteins. Although IFNγ has been reported to induce oxidative damage in mouse models of inflammation, such as EAE (Espejo et al., 2002), the mechanism for this oxidative stress is not clear and could be through macrophage activation, neutrophil recruitment, or other complex mechanisms. Induction of iNOS by IFNγ results in protein nitration rather than protein carbonylation, and the oxidative damage that Seifert et al. (2010) documented was with the Oxyblot method, which detects carbonyl groups on proteins and not protein nitration.

Finally, these observations show that ISG15, a ubiquitin-like protein that is induced by IFN α and β, is also significantly induced by IFNγ. Although we did not identify ISGylated proteins, the induction of the E2 for ISGylation (Ube2L6) and the E3 (Herc5) by IFNγ strongly suggests that some cell proteins are likely to be modified by formation of ISG15 conjugates. Indeed, ISGylation of newly transcribed proteins can inhibit virus production (Durfee et al., 2010), but no evidence has been obtained that ISG15 is promoting proteasomal degradation and/or MHC class I restricted presentation. In summary, although immunoproteasomes have a clear role in the generation of MHC class I peptides and in cytokine induction (Muchamuel et al., 2009), they do not preferentially degrade polyubiquitinated proteins, remove ubiquitin-containing inclusions, or significantly alter the progression or severity of experimental autoimmune encephalomyelitis.

## Experimental Procedures

### Cell Lines, Tissue Culture, and Mice

HeLa cells were cultured in DMEM medium under standard conditions. The murine fibroblast cell line B8 (Groettrup et al., 1995) and mouse embryonic fibroblasts (MEF), prepared from C57BL/6 mice and *LMP7*^−*/*−^ mice (Fehling et al., 1994), were cultivated under standard conditions and kept in IMDM or DMEM-medium. Mice were kept in a specific pathogen-free facility and used at 6–10 weeks of age. Animal experiments were approved by the review board of Regierungspräsidium Freiburg.

### Western Blotting and IFNγ Stimulation

HeLa cells were stimulated with 100 U/ml IFNγ for the indicated time points. The cells were then lysed by sonication in 25 mM HEPES pH 7.4, 1 mM ATP, 1 mM DTT, 5 mM MgCl_2_, and 10% glycerol. Protein concentration of the lysates were determined using the Bradford reagent (Pierce) and equal amounts of protein were separated by SDS PAGE and analyzed by western blotting. The murine B8 cells and MEFs were stimulated with 200 U/ml murine recombinant IFNγ (Peprotech) for the indicated time points and lysed either in 20 mM TRIS-HCl, pH 7.5, 10 mM EDTA, 100 mM NaCl, 1% NP40, 10 μM MG-132, 5 mM NEM, and Complete Protease Inhibitor Cocktail (Roche) (Figure 1B), or in 10 mM Tris, 150 mM NaCl, 1% Triton X-100 (Figure 4C) and the amount of protein was determined with a Pierce BCA protein assay kit (Thermo scientific). Western blot analysis was performed in the usual manner.

### Purification of 26S Proteasomes from Mouse Tissues

26S particles were purified from muscles and spleens of CD1 mice by the Ubl-affinity method in the presence of 150 mM NaCl (Besche et al., 2009). Protein content was measured by Bradford assay and the levels of pure proteasomes in the different tissues were normalized to the levels of the constitutive subunits, Rpt1, Rpt5, and the 20S α subunits, determined by western blot densitometry evaluation (ImageJ quantification).

### Measurements of Proteasome Activity

Suc-LLVY-AMC, Suc-GGL-AMC, Suc-LLR-AMC, and Ac-nLPnLD-AMC were purchased from Bachem. Cell lysates (5 μg) were incubated with the peptides in a buffer containing 50 mM Tris pH 7.4 with 40 mM KCl, 5 mM MgCl_2_, 1 mM ATP, and 1 mM DTT. Kinetic fluorescence was measured in triplicate in a 96-well format (Molecular Devices). The fluorescence was normalized to AMC levels by first generating a standard curve for AMC fluorescence. The lysates were also treated with 1 μM Bortezomib to inhibit specifically the proteasome’s peptidase activity. The low amount of Bortezomib-resistant activity in the lysate was then subtracted from the total peptidase activity, so that only the proteasome-specific fluorescence was measured. The peptidase activity of the pure proteasomes was measured, as described using 2 nM of pure proteasomes.

Proteasomal degradation of a polyubiquitinated protein was measured using Ub_5_DHFR as the substrate. Ub_5_DHFR (a kind gift of Millenium Pharmaceuticals) was radiolabeled as described previously with some modifications (Lam et al., 2005). Ub_5_DHFR (10 μg) was incubated with 20 U PKA, 50 μCi ATP[^32^P], 1 μg BSA at 37°C for 15 min in a buffer containing 20 mM Tris pH 7.5, 100 mM NaCl, 10 mM MgCl_2_, and 1 mM DTT. The nonlabeled Ub_5_DHFR was removed by passing the reaction through a BioRad MicroSpin column. Isolated mammalian proteasomes (2 nM) were then incubated with 30 nM of the ^32^P-Ub_5_DHFR for up to 30 min in 50 mM Tris pH 7.4, 5 mM MgCl_2_, 2 mM ATP, 1 mM DTT, and 0.0 2mg/BSA (total reaction volume 30 μl). Twenty microliters of the reaction was then added to 20 μl of ice cold BSA (10 mg/ml), and the proteins precipitated by adding 34 μl of ice cold 40% TCA. The samples were incubated on ice for 10 min and centrifuged at 13,000 rpm for 10 min. Supernatants (20 μl) were added to 4 ml of scintillation fluid, and the acid soluble counts measured.

### Immunocytochemistry

MEFs from C57BL/6 wild-type or *LMP7*^*−/−*^ mice were grown on coverslips in 24-well plates and treated with 200 U/ml IFNγ (Peprotech) for the indicated times. Cells were fixed for 20 min with 4% paraformaldehyde and permeabilized with 0.2% Triton X-100 for 10 min at room temperature and stained with an anti-ubiquitin monoclonal antibody FK2 (ENZO, dilution 1:500). Cells were first labeled with primary antibodies, followed by washing and incubation with the respective Alexa Fluor-labeled secondary antibody (F(ab)2) (dilution 1:400, Invitrogen). All antibodies were diluted in 0.2% gelatin. All incubations were carried out for 1 hr at room temperature. Images were analyzed with an LSM510 confocal laser-scanning microscope (Carl Zeiss) using a 63× plan-apochromat, oil-immersion objective (NA = 1.4). Pictures were analyzed with ImageJ software. To quantify the “ALIS,” random fields of at least 70 cells were stained for ubiquitin with the FK2 mAb, and the number of ALIS/cell was assessed by counting extranuclear, ubiquitin positive structures larger than 0.5 μm^2^. The number of ALIS per cell was calculated by dividing the total area of FK2 fluorescence of all ALIS per cell by the minimum ALIS-size of 0.5 μm^2^. Statistical analysis was performed for three independent experiments (N > 210 cells).

### Induction of EAE

C57BL/6 mice were immunized subcutaneously in the lateral abdomen with 200 μg MOG_35–55_ peptide in CFA, and pertussis toxin (200 ng) in PBS was administered on day 0 (intraperitoneally [i.p.]) and day 2 (i.p.). The synthetic peptide MOG_35–55_ (MEVGWYRSPFSRVVHLYRNGK) was obtained from GenScript (Piscataway, NJ). Disease symptoms were scored as indicated in the legend to Figure 5.

## Supplemental Information

Document S1. Article plus Supplemental Information
